# Development and Implementation of Survivorship Tools to Enable Medical Follow-Up After Childhood Cancer Treatment in Southern Sweden

**DOI:** 10.1200/CCI.18.00130

**Published:** 2019-06-07

**Authors:** Magnus Petersson-Ahrholt, Thomas Wiebe, Lars Hjorth, Thomas Relander, Helena M. Linge

**Affiliations:** ^1^ Climber Sweden, Stockholm, Sweden; ^2^ Lund University, Lund, Sweden; ^3^ Skåne University Hospital, Lund, Sweden

## Abstract

**PURPOSE:**

Survival rates after childhood cancer have increased from 20% to 80% since the 1970s. The increased number of survivors emphasizes the importance of late effects and their monitoring. Late effects may have a strong impact on quality of life in survivors. The purpose of this study was to make key data in a quality registry available for direct clinical use, enabling health care professionals to perform efficient and appropriate long-term medical follow-up after childhood cancer treatment.

**METHODS:**

The population-based quality registry upon which this study is centered contains data on all individuals diagnosed with childhood cancer (diagnosed at 18 years of age or younger) in southern Sweden since January 1, 1970, and treatment data on 5-year survivors. Web tools, which were developed and implemented in a health care setting, generate a personalized treatment summary for each patient and enable risk group stratification of survivors.

**RESULTS:**

Generation of a personalized treatment summary and risk group stratification of survivors led to identification of women at risk for developing breast cancer as a consequence of childhood cancer treatment. Three novel cases of previously undiagnosed breast cancer were identified.

**CONCLUSION:**

The registry, together with the developed tools, enabled health care professionals to perform medical follow-up in this at-risk patient population.

## BACKGROUND AND SIGNIFICANCE

Cancer treatments for children and adolescents are complex and disease specific. Treatment includes a panel of therapeutic approaches, eg, surgery, chemotherapy (including new-generation drugs such as antibody-based therapies and small molecules such as tyrosine kinases), radiotherapy, and stem-cell transplantation. Since the 1970s, long-term survival rate after childhood cancer has increased from 20% to 80%. The increased survival rate is a success story, but the increasing number of survivors (approximately 11,000 survivors in Sweden and 300,000 to 500,000 in Europe) emphasizes the issue and extent of late effects. These may have a strong impact on the health and quality of life of survivors as they transition to adulthood and throughout the rest of their lives, with significant costs to society as well as the individual.^1^

In recent years, several European Union–funded initiatives (European Network for Cancer Research in Children and Adolescents, PanCare Childhood and Adolescent Cancer Survivor Care and Follow-Up Studies, and European Expert Paediatric Oncology Reference Network for Diagnostics and Therapeutics) have supported actions to address the multifaceted problems that are associated with survivorship. Late effects may become evident several decades after childhood cancer treatment has ended. Hence the underlying cause of the late effect may not be clear to the individual, and details of the individual’s treatment history may be cumbersome or impossible to obtain. Global efforts by organizations such as the International Guidelines and Harmonization Group and the PanCare Childhood and Adolescent Cancer Survivor Care and Follow-Up Studies consortium have resulted in evidence-based guidelines for long-term medical follow-up after childhood cancer treatment. To date, guidelines for breast cancer screening, cardiotoxicity, male and female fertility, and thyroid cancer surveillance have been finalized.^2-6^

## OBJECTIVES

The overall aim of the study was to make data from a quality registry available for direct clinical use and to provide patients and health care professionals with correct and secure information to enable long-term follow-up. Objectives of this study were as follows: 1) to create a clinically relevant treatment summary for childhood cancer survivors on the basis of their treatment history as entered into the regional quality registry BORISS (BarnOnkologiskt Register I Södra Sjukvårdsregionen [a pediatric oncology registry in southern Sweden]),^7^ and 2) to create a tool for risk group stratification of the survivors for health care use.

**CONTEXT**

**Key Objective**
To make key data in a population-based quality registry available and presentable for direct clinical use in a clinic that specialized in the late effects in childhood cancer survivors.
**Knowledge Generated**
Application of tools to a population-based quality registry enabled a data-driven approach to survivorship care of patients treated for cancer during childhood. It supported the launch of an intensified screening program that identified undiagnosed breast cancer cases in at-risk survivors. This example of digitization of a data source other than the medical chart supported recommended risk-based, survivor-focused care.
**Relevance**
Adequate use of the quality registry through the developed tools that enabled presentation of a personalized treatment summary and risk group stratification centered on evidence-based guidelines to optimize risk-directed survivorship care of those treated for cancer during childhood.

## METHODS

**FIG 1.**
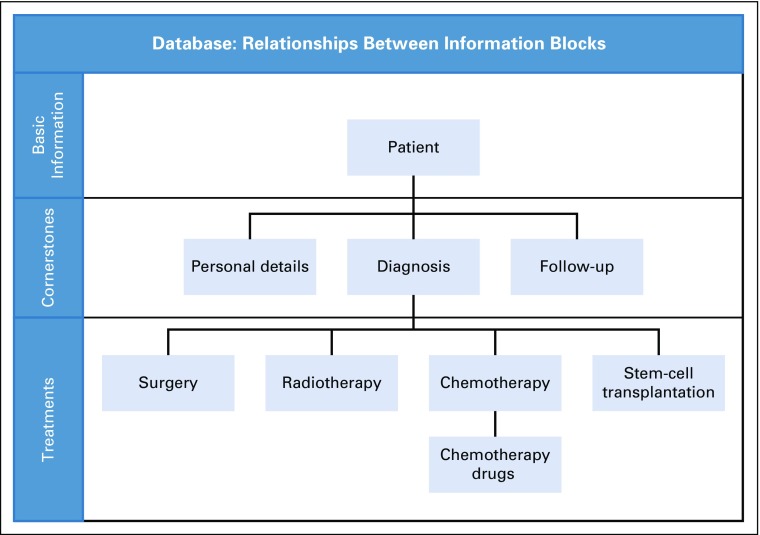
Information structure of the registry. The basic information in the registry concerns the patient. The cornerstones concern the diagnosis (personal identification number, diagnosis, histology, localization, and identity of pathologic report) and personal details (personal identification number, sex, age, place of residence, and vital status), which are collected from the national cancer registry and the national population registry, respectively. The treatment information, which is abstracted from medical charts, is entered into the registry via a Web-based form.

In 2005, a regional quality registry for all pediatric cancer cases (patients who were 0 to 18 years of age at diagnosis) occurring in southern Sweden with a diagnosis date after January 1, 1970, was initiated by experienced pediatric oncologists (with more than 35 years of clinical experience). To date, the population-based registry contains data on all children diagnosed with cancer between January 1, 1970, and December 31, 2016 (N = 2,928). Data are imported annually from the national population registry and the national cancer registry. The personal identification number, sex, age, and place of residence are variables supplied by the national cancer registry and concern the basic information and cornerstones in the registry data (Fig 1). Medical data supplied include tumor site (ICD-7, ICD-9, ICD-O2, or ICD-O3, depending on era of diagnosis) and histologic type (C24, ICD-O2, or ICD-O3). The basis of diagnosis, the date of diagnosis, the reporting hospital, department and pathology/cytology department, as well as the identification number for the tissue specimen, are supplied. The cornerstone “follow-up” is in place but does not contain data. The variables of personal identification number, sex, first and last name, vital status, permanent residence, and basis of diagnosis are loaded without transformation. Typically dates such as date of diagnosis or date of death are transformed to Structured Query Language–appropriate format smalldatetime, and time periods such as age at death are transformed by calculation using other available values. Medical treatment data (Fig 1, blue boxes) are abstracted from medical records, and earlier coding is translated into ICD-10 and ICD-O3. All data are then entered into the database through a Web interface. The BORISS registry resides within and is the property of the county of Skåne. The county of Skåne has authorization to gather data from adjacent councils of Halland, Kronoberg, and Blekinge, which together comprise southern Sweden (population of 1.8 million). The registry has been described previously.^7^

To enable clinical use of data in the registry, we applied a Web tool interface (based on Qlikview) to data to generate a simplified, clinically relevant treatment summary through an application called ZAPP. We used the anthracycline dose equivalents put forward in national guidelines. The POP application uses the same registry data as ZAPP but with a different purpose. The application uses an associative model with in memory technology, which makes all dimensions and measures searchable by the user. The user can filter the results on any of the dimensions and measures available, which enables dynamic selections of risk groups as new guidelines and research become available. The associative model enables the user to quickly see how data are connected or not connected. For instance, the user selects a particular diagnosis and retrieves information on what chemotherapeutic drugs were present or absent in the treatment regimens. This enables the user to make selections on the basis of evidence-based risk criteria and retrieve the contact information of individuals to enable medical follow-up. Selections and groupings made by the user can be saved and shared with others. Both applications use BORISS data with transformations done to maximize usability and apply mapping structures that are not available in underlying data. An example is the presentation of cumulative doses of the chemotherapeutic drugs, which is important for assessing risks of certain late effects.

## INFORMATION SECURITY ISSUES

Users of the applications can only view and analyze data, but they can introduce labels on the basis of the associative data model found in the software. Use of the applications within the health care setting is limited by two-factor authentication. Only one patient treatment summary can be viewed at a time in ZAPP. To reflect each individual’s risks, the application relies on dynamic calculations of the treatment detail levels such as radiation or chemotherapy dose received. The application underwent four rounds of data verification to ensure correct data presentation, correct calculations of anthracycline equivalencies and cumulative chemotherapy doses, and adequate usability.

## RESULTS

### ZAPP as a Tool for Creating an Individualized Historic Treatment Report

**FIG 2.**
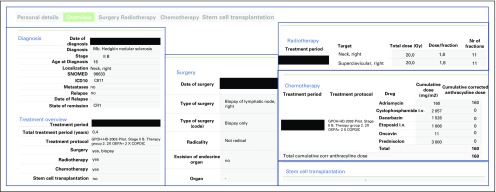
Example of patient treatment summary. The detailed patient treatment summary contains the headings “Personal details”, “Diagnosis”, “Overview”, “Surgery”, “Radiotherapy”, “Chemotherapy”, and “Stem-cell transplantation”. For reasons of privacy, personal details are excluded in the example, and the other information is anonymized by black boxes.

The summary of each individual’s complete treatment (Fig 2) was presented and made digitally accessible to health care professionals in the pediatric oncology unit and at the Late Effects Clinic at Skåne University Hospital in Lund, Sweden. Relevant risk group designations in the form of visual flags for specific treatment characteristics are included on the relevant summaries. The summary can currently be obtained by the survivor through contact with health care services either in the transition between pediatric and adult care or at the Late Effects Clinic. Providing the patient with direct digital access is in progress.

### POP Risk Group Stratification of the Registry Population

POP enables a designated nurse specialist to filter the registry population of survivors according to risk group categories (Table 1), which leads to contact for medical follow-up. It also allows continuous inclusion of individuals who meet risk group criteria as they transition from pediatric to adult care (in Sweden, at 18 years of age). In April 2016, we performed a risk group–based outreach to women at risk for either developing breast cancer as a second malignancy or suffering from cardiac late effects of childhood cancer treatment. Through the filtering application, we identified 48 female childhood cancer survivors, who were older than 25 years, were 8 years postdiagnosis, and who had received radiation to the chest (10 to 19.99 Gy or more than 20 Gy), either alone or in combination with anthracycline (any dose) treatment. These women were called upon to contact health services at the Late Effects Clinic at Skåne University Hospital in Lund, Sweden. Two women had already been diagnosed and treated for breast cancer. Among the remaining, two novel cases of breast cancer were identified through an intensified screening program (annual mammograms and/or magnetic resonance imaging), and as a consequence of the raised awareness, one additional case was identified in 2017.

**TABLE 1.**
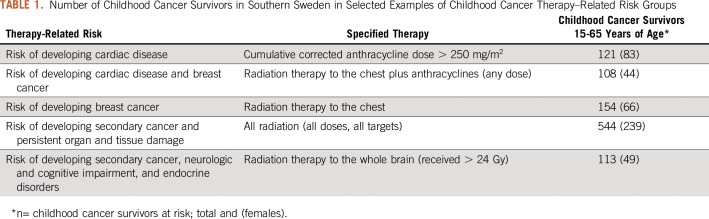
Number of Childhood Cancer Survivors in Southern Sweden in Selected Examples of Childhood Cancer Therapy–Related Risk Groups

The registry-associated applications (ZAPP and POP) have been implemented at the Late Effects Clinic at Skåne University Hospital in Lund, Sweden.

## DISCUSSION

The advances in treatment of childhood cancer have increased survival to 80% and generated a large increase in the number of survivors. The severity of late effects associated with early treatment protocols has been reduced in later years as the result of efforts made toward more focused and risk-adapted treatments.^8^ The strategy presented herein facilitates recommended risk-based survivor-focused care and builds on key points of the underlying registry, namely that it is population based, it serves multiple purposes depending on the tools applied, and its basis on mandatory registration in the national cancer registry. The ability to implement survivorship tools, as we have done, depends on having access to detailed treatment data in a structured digital form and permission of regional authorities to put it to clinical use. Another prerequisite for implementation is that the registry goes back to 1970, because late effects may not surface until several decades have elapsed. The Swedish Data Protection Authority does not allow medical data of Swedish citizens to leave the country, which excludes cloud storage that is owned by a foreign entity. From the perspective of patients and health care professionals, the concept of access to treatment history and medical follow-up is essential to ensure that appropriate steps are taken to improve and maintain quality of life, limit suffering, reduce health care costs, and ultimately prevent premature loss of life. Development of the treatment summary allows future studies to address what impact the summary may have on quality of life in childhood cancer survivors.

The current approach represents one of the strategies used to optimize risk-directed survivorship care. Local rules apply for access and use of health care data, but the clinical need for long-term follow-up has given rise to multiple solutions,^9,10^ all of which benefit adherence to guidelines, communication with survivors, and awareness of late effects.

A large-scale European technical solution, the Survivorship Passport, builds on available guidelines and recommendations of medical follow-up and is a backbone for gathering and presenting patients’ comprehensive childhood cancer treatments.^11^ Treatment details are entered into a Web-based form, which triggers recommendations of medical follow-up. The time-consuming extraction step from medical charts, together with the manual entry of data by a health care professional, remains a problem for a work force often burdened with competing demands.

An Austrian survivorship tool works by directly accessing electronic health care records and adding algorithms, which trigger medical follow-up recommendations resulting in a personalized report. Entry of childhood cancer treatment into a designated part of the health care record is mandated by law, obliterating the need for extra registration steps. Because of its agile online-based data retrieval, the Austrian implementation may be more adaptable as guidelines change or additional knowledge is gained. Implementation started in 2017 and benefits patients who were recently cured. For patients who were treated in previous decades, another solution must still be applied.

In Sweden, the national registry for late effects after childhood cancer treatment is a collaboration between the six childhood cancer treatment centers. Data in this registry have partial coverage, particularly for historic cases, and the registry lacks data on radiation targets. The clinical need to initiate screening processes and facilitate the work at the Late Effects Clinic warranted proceeding with putting the regional registry BORISS to clinical use. Through implementation of the filtering tool in the health care setting, we were able to identify previously unknown cases of breast cancer at an early stage. The POP application enables dynamic selection of risk groups by the user as new guidelines are published or new research results become available. Other actionable risk groups include those at risk for cardiac toxicity as the result of treatment with high-dose anthracyclines alone or in combination with chest radiation, which may result in premature deaths.

In addition to its clinical use, the BORISS registry continues to provide data for research purposes after ethical approval for each respective study.^12-16^

## CONCLUSION

Two digital survivorship tools centered on a population-based quality registry were developed and implemented in a publicly funded health care setting. The current work represents a step toward digitalizing health care.

### Clinical Relevance Statement

Sufficient knowledge of each individual’s treatment history after childhood cancer is crucial to determining the risk for late complications. By applying business intelligence software to a quality registry, significant progress has been made in providing appropriate medical attention to this group.

### Human Subjects Protections

In agreement with the laws governing quality registries, information was collected to monitor and enhance the quality of care provided at Skåne University Hospital. The law does not impose a requirement of consent from the individual. At the time of diagnosis, the parents of patients were informed and presented with the possibility of opting out from several quality registries. All activities were approved by the regional ethical review board and the hospital governing board in Lund, Sweden.^17^
